# An investigation of the presence and antimicrobial susceptibility of *Enterobacteriaceae* in raw and cooked kibble diets for dogs in the United Kingdom

**DOI:** 10.3389/fmicb.2023.1301841

**Published:** 2024-01-08

**Authors:** Genever Morgan, Gina Pinchbeck, Eda Taymaz, Marie Anne Chattaway, Vanessa Schmidt, Nicola Williams

**Affiliations:** ^1^Institute of Infection, Veterinary and Ecological Sciences, University of Liverpool, Neston, United Kingdom; ^2^Gastrointestinal Bacteria Reference Unit, United Kingdom Health Security Agency, London, United Kingdom

**Keywords:** raw, dog, food, *Enterobacteriaceae*, antimicrobial resistance, pet, *E. coli*

## Abstract

Raw meat diets (RMD) for dogs are an increasingly popular alternative pet food choice, however studies worldwide have demonstrated them to be contaminated with zoonotic and antimicrobial resistant (AMR) bacteria, including bacteria resistant to critically important antibiotics. Despite this, few data exist surrounding the presence of these bacteria in RMD in the United Kingdom. The present study aimed to identify the most commonly selected RMD and non-raw diets (NRMD) by United Kingdom dog owners. Additionally, it investigated the presence of AMR-*Enterobacteriaceae* in samples of pre-prepared RMD and cooked commercial kibble dog foods. An online survey investigating diet preferences of United Kingdom dog owners was open for 6 weeks between February–March 2020. From this, the top 10 brands of pre-prepared raw and cooked kibble diets were ascertained and 134 samples purchased (110 RMD, 24 kibble) and subjected to microbiological testing. Bacterial enumeration of *E. coli* and other *Enterobacteriaceae* was undertaken, and the presence of *Salmonella* spp. and AMR-*E. coli* within samples determined. Whole genome sequencing was undertaken on *Salmonella* spp. and third-generation cephalosporin-resistant 3GCR-*E. coli* isolates. Pre-prepared RMD was most commonly selected by dog owners who fed RMD, and cooked commercial complete dry food was most frequently fed by owners who fed NRMD. Damaged and leaking packaging was observed in samples of RMD, alongside variability in information provided surrounding product traceability. Counts of *E. coli* and other *Enterobacteriaceae* exceeding >5,000 CFU/g were identified in samples of RMD. AMR-, extended-spectrum beta-lactamase (ESBL)-producing and 3GCR-*E. coli* was isolated from 39, 14 and 16% of RMD samples, respectively. Multiple antimicrobial resistance genes were identified in 3GCR-*E. coli* isolates. Of the ESBL encoding genes, *bla*_CTX-M-15_ was most commonly identified. *S. enterica* was isolated from 5% of RMD samples. No *Enterobacteriaceae* were isolated from any of the cooked kibble samples. The present study suggests that pre-prepared RMD available for dogs in the United Kingdom can be contaminated with zoonotic and AMR-*Enterobacteriaceae*. RMDs, therefore, are potentially an important One Health concern. Veterinary and medical professionals, pet food retailers and pet owners should be aware of these risks; and stringent hygiene measures should be practiced if owners choose to feed RMD.

## Introduction

There is a diverse range of diets currently available for dog owners to select from to feed their pets, and while conventional cooked proprietary diets (including dry and semi-moist kibbles, tins and trays of wet food) make up the majority of pet dog diets, there is increasing availability of alternative options such as home-made diets, vegetarian/vegan foods, insect-based and raw meat-based diets (RMD). Indeed, many dogs are fed diets where non-conventional options comprise some or all of the ration (Dodd et al., 2020).

RMD are an increasingly popular diet choice. Broadly, RMD are composed of uncooked animal-derived material including muscle, internal organs, bones, skin and tendons (Freeman et al., 2013). The 2022 PDSA Animal Welfare (PAW) report that surveyed a sample of dog owners which was demographically representative of the United Kingdom population, stated that 7% of dogs in the United Kingdom were fed RMD, with 5% fed pre-prepared RMD and 2% fed a home-made or home-prepared diet, although the true prevalence within the United Kingdom dog-owning population may be higher (PDSA, 2022). Additional data, such as market share, are limited regarding the actual proportion of RMD fed to dogs in the United Kingdom. However investigation of the Animal and Plant Health Agency (APHA) surveillance data from 2008 to 2018 indicated that the number of production plants registered to produce RMD increased greatly in that time period suggesting a response to increased demand (Withenshaw et al., 2020).

Raw materials which are classed as category 3 animal by-products by the Department for Environment and Rural Affairs (Defra) are allowed to be utilized in RMD for pets (Defra/APHA, 2018) This may include meat and carcasses passed fit for human consumption at the slaughterhouse, and animal material originally intended for human consumption but rejected for commercial reasons. It can also include material from animals which passed an antemortem inspection but was subsequently deemed unfit for human consumption (Defra/APHA, 2019). Regular product sampling must be undertaken for both *Enterobacteriaceae* and *Salmonella* spp. (Defra/APHA, 2020).

A variety of bacterial pathogens have been isolated from raw pet food worldwide including *Escherichia coli*, *Salmonella* spp., *Campylobacter* spp., *Listeria* spp. and *Clostridium* spp. (Weese et al., 2005; Strohmeyer et al., 2006; Mehlenbacher et al., 2012; Nemser et al., 2014; Bojanić et al., 2017; Van Bree et al., 2018; Hellgren et al., 2019; Bottari et al., 2020; Kananub et al., 2020; Treier et al., 2021). In the United Kingdom there have been limited studies specifically investigating the presence of bacterial pathogens in RMD, however there have been a number of recalls involving RMD reported by the Food Standards Agency due to the presence of *Salmonella* spp. in particular (Food Standards Agency, 2021a,b, 2022, all accessed March 2023). The number of *Salmonella* spp. isolations associated with raw pet food increased up to the year 2018, and this has been linked to a concurrent increase in the number of plants registered to produce RMD (Withenshaw et al., 2020). Between 2014 and 2018, the number of *Salmonella* spp. isolations from RMD sampled by the APHA ranged from 26 to 244 isolations per year, compared to 4–27 isolations per year for cooked commercial kibble-based food, with the highest number of isolations occurring in both RMD and cooked kibble in 2018 (Withenshaw et al., 2020). In addition, in 2017 a cluster of human cases of Shiga-toxin producing *E. coli* O157:H7 in the United Kingdom was epidemiologically linked to the provision of contaminated RMD containing tripe (Kaindama et al., 2020).

*Escherichia coli* makes up part of the normal mammalian commensal intestinal flora (Johnson and Russo, 2002), and as such, is utilized as an indicator of fecal contamination of food products (Strohmeyer et al., 2006). The EU absolute threshold for numbers of *E.coli* in raw pet food is 5 × 10^3^ CFU/g [Commission Regulation (EU) No 142/2011] at the point of production, however numbers in raw pet food samples in Europe commonly exceed this (Davies et al., 2019). RMD products are often described as comprising ‘human grade’ meat which may lead to the perception of a better microbiological quality. However, an Italian study which sampled raw meat pet diet products (*N* = 112) which were of ‘human grade. but no longer intended for human consumption due to defects, manufacturing problems or commercial reasons, identified the presence of *E. coli* in 100% (*N* = 52) of poultry samples, 100% (*N* = 30) of pork samples and 93% (*N* = 28) of beef samples tested (Bacci et al., 2019), as well as *Salmonella* spp. in 12% (*N* = 6) of poultry and 13% (*N* = 4) of pork samples.

Alongside the zoonotic disease concerns, there is increasing interest surrounding the potential for raw pet foods to be a source of antimicrobial-resistant (AMR) bacteria. Of particular interest is the presence of transmissible extended-spectrum beta lactamase (ESBL)-producing, and third generation cephalosporin resistant (3GCR), *Enterobacteriaceae.* Such resistances are of concern; not only do they confer resistance to beta-lactam antibiotics, but also hydrolyse third generation cephalosporins including cefotaxime, ceftiofur, cefpodoxime and ceftazidime, which are highest priority critically important antibiotics (WHO, 2019), and are increasingly associated with multidrug resistance (Livermore and Hawkey, 2005; Livermore, 2008; Wedley et al., 2017). A high prevalence of ESBL-producing and 3GCR-*Enterobacteriaceae* has been reported in pre-prepared RMD in European studies (Nilsson, 2015; Van Bree et al., 2018; Nüesch-Inderbinen et al., 2019), as well as from meat products previously intended for the human food chain, but destined for pet food production (Bacci et al., 2019). Furthermore, RMD-feeding has been demonstrated to be a risk factor for canine fecal shedding of AMR-bacteria (Van den Bunt et al., 2020), and studies from the United Kingdom and Sweden observed that dogs fed RMD were significantly more likely to shed AMR-*E. coli* than those fed conventional cooked diets (Runesvärd et al., 2020; Groat et al., 2022).

Despite the interest in alternative diet choices and the growing canine and public health concerns regarding RMD, there are few studies investigating this aspect of dog ownership in the United Kingdom. Importantly, despite studies globally identifying microbiological and AMR risks associated with RMD products, currently there are no data surrounding pre-prepared RMD available for dogs in the United Kingdom. The aims of the present study were firstly to identify the most common RMD and non-raw meat-based diets (NRMD) selected by United Kingdom dog owners, their preferred treat types and from where owners obtained their dog’s food. Secondly the study aimed to investigate the prevalence of *E. coli*, other *Enterobacteriaceae* and *Salmonella* spp. within both commonly-purchased pre-prepared RMD and NRMD, as well as the prevalence of AMR- and ESBL-producing *E. coli* within the most commonly fed diets.

## Materials and methods

An online survey titled ‘A Dog’s Dinner: A survey investigating dog food selection by United Kingdom dog owners’ was created using JISC online software. The survey was available to dog owners from the whole of the United Kingdom, regardless of dog food preference and some findings including participant demographics, as well as methods of dissemination and participant recruitment, have been published (Morgan et al., 2022). The survey was advertised via social media, at Crufts Dog Show 2020 and via letters to veterinary news publications, and was available online for approximately 6 weeks from the 19th of February to the 31st of March 2020.

A sub-section of the questionnaire involved questions specifically regarding the diet fed, including the types of food chosen, the sources from where foods were obtained, the preferred meat types (RMD only), preferred treat types and the preferred brands chosen. Dog owners were directed to either a raw-feeding or non-raw feeding specific set of questions, depending on their answer to the question “Do you feed any raw animal material to your dog(s).” Owners were requested to complete this section once on behalf of all dogs in the household if they were fed the same diet, or individually for each dog that was fed differently, up to a possible total of 10 dogs per owner.

Ethical approval was granted by the University of Liverpool Veterinary Research Ethics Committee (approval number VREC913).

## Statistical methods

The sample size of participants required to achieve statistical power for the online survey was calculated to be 1,066, using an estimated prevalence of raw feeding of 50%, with 3% precision.

Analysis was undertaken using SPSS 27 [IBM Corp. (released 2020). IBM SPSS Statistics for Windows, Version 27.0. Armonk, NY: (IBM Corp.). The frequency and percentage] of responses from participants feeding either RMD or NRMD were computed. RMD were classed as those including raw animal material more than once weekly, and NRMD were classed as all diets comprising cooked material (e.g., kibble, cans, trays and sachets of cooked commercial wet food, home cooked diets, vegetarian diets, etc).

Descriptive analysis (frequency, percentage) was undertaken for both raw and non-raw food choices. Type of food preferred, source of food and types of treats were compared, and included options provided in the survey and those identified in the free text answers provided by owners. In addition, sources of non-pre-prepared raw meat were determined. Finally, the top 20 most frequently utilized brands of pre-prepared RMD and of NRMD were identified from the free text answers provided by dog owners.

## Sampling methods

The 10 most frequently utilized brands each of RMD and NRMD identified from the survey were sampled. Where a RMD brand was not readily available for purchase (due to reasons such as lack of availability, inability to courier small order sizes, or availability only as a subscription service), another brand from the top 20 was selected instead. Samples (RMD 9–15 samples per brand, total 110 samples, NRMD 1–3 samples per brand, total 24 samples) were purchased approximately monthly, one brand at a time, directly from their brand websites (RMD) or from pet shops online and in person (NRMD) between August 2020–October 2021. Sample flavors were selected based on availability at the time of purchase, and to reflect a range of meat types available per brand. All RMD samples were pre-prepared items and were supplied frozen. All NRMD samples were cooked kibble.

### Bacterial culture and identification

Each food sample was assigned a unique number, and the brand, sample type (RMD, NRMD), batch number/lot code (where present), country of origin of ingredients and whether the product was produced in the United Kingdom was recorded. Sample packets were inspected for packaging material type and any evidence of damage or leakage. To ensure no cross-contamination of samples, RMD samples were stored frozen as per manufacturer instructions and defrosted fully in a refrigerated unit prior to testing within separate containers. NRMD samples were stored at room temperature and bags were opened only at the time of sampling. All samples tested were used within the ‘use-by’ date where this was provided. Three brands did not have a ‘use-by’ date provided, however all samples were tested within 1 week of their delivery to the laboratory.

The amount of food (25 g) to be tested was collected aseptically using sterile instruments from multiple sites within the food sample, and homogenized via stomaching in a sterile plastic stomaching bag for 1 min with 225 mL of buffered peptone water (BPW), at room temperature. Approximately 20 mL of homogenate was poured into a sterile universal tube and incubated aerobically at 37°C for 18 ± 2 h. The remainder of the RMD sample was placed into a sterile sealable bag and repeat frozen at -20°C for further testing at a later date if required. NRMD bags were re-sealed and stored at room temperature.

Following incubation, a 5 μL loopful of BPW homogenate was used to inoculate one each of a chromogenic Harlequin *E. coli*/Coliform Agar (HECA) (Neogen, United Kingdom) and a HECA plate infused with 1 μg/mL cefotaxime (HECA+Cx), and incubated overnight at 37°C. Further homogenate (100 μL) was added to 10 mL of Rappaport Vasiliadis Broth (RVB) and incubated overnight at 42°C for *Salmonella* species culture.

Following incubation, the HECA plates were analyzed for the presence of typical *E. coli* colonies (dark blue-violet, 0.1 mm-2 mm diameter), and four such colonies were picked and plated onto nutrient agar (NA) (Neogen, United Kingdom). The HECA+Cx plates were analyzed for both typical blue *E. coli* and rose-pink colonies indicative of other *Enterobacteriaceae* (e.g., *Enterobacter* spp.) and 2 colonies of each (if present) were picked and plated onto NA. One 5 μL loopful of the incubated RVB was plated to Harlequin Chromogenic Agar for Salmonella Esterase (CASE) (Neogen, United Kingdom). The NA and CASE plates were incubated for 18 ± 2 h at 37°C.

Isolates which were phenotypically identified as *E. coli* underwent PCR for the *uspA* gene to confirm them as *E. coli* prior to undergoing whole genome sequencing. Methods were as per Anastasi et al. (2010). Primers used were CCGATACGCTGCCAATCAGT (forward) and ACGCAGACCGTAGGCCAGAT (reverse), with an amplicon size of 884 base pairs.

Following incubation, the CASE plates were analyzed for the presence of suspected *Salmonella* spp. (turquoise blue/green colonies) and if present, two colonies were picked and plated onto NA before overnight incubation at 37°C. Confirmation of *Salmonella* spp. was then undertaken via matrix-assisted laser desorption/ionization-time of flight mass spectrometry (MALDI-TOF).

The *E. coli* isolates from plain HECA plates, and *Salmonella* spp. isolates, underwent antimicrobial susceptibility testing (AST) via the disk diffusion method using seven antibiotic disks chosen based on European Committee on Antimicrobial Susceptibility Testing (EUCAST) recommendations (EUCAST, 2022). Isolates were inoculated into sterile saline using a 5 μL loop to 0.5 McFarland units then a sterile swab was used to spread the inoculated saline onto Muller-Hinton agar (Neogen, United Kingdom) and antibiotic disks were applied. Plates were incubated aerobically at 37°C for 18 ± 2 h. Antimicrobials tested were ampicillin 10 μg, amoxycillin-clavulanic acid 20 μg/10 μg, ciprofloxacin 5 μg, tigecycline 15 μg, trimethoprim-sulphamethoxazole 1.25 μg/23.75 μg, amikacin 30 μg and meropenem 10 μg (MAST Group Ltd., Liverpool United Kingdom). A susceptible control strain of *E. coli* (ATCC 25922) was also tested to ensure disk efficacy.

Following incubation, zones of inhibition (ZOI) for each antibiotic disk were measured to the nearest millimeter. EUCAST clinical breakpoints (EUCAST, 2022) were used for interpretation for all antibiotics other than amoxycillin-clavulanic acid, where the breakpoint used for interpretation was as recommended by the Clinical and Laboratory Standards Institute (2020).

Multidrug resistance (MDR) was defined as demonstrated phenotypic resistance to three or more classes of antibiotics tested (Magiorakos et al., 2012).

The *E. coli* isolates from HECA+Cx plates initially underwent extended-spectrum beta-lactamase (ESBL) double-disk test using paired disks of cefotaxime 5 μg and cefotaxime 5 μg + clavulanic acid 10 μg, and ceftazidime 10 μg and ceftazidime 10 μg + clavulanic acid 10 μg (EUCAST ESBL detection set, MAST Group Ltd., Liverpool United Kingdom). Plates were incubated at 37°C for 18 ± 2 h. Isolates which were deemed ESBL-positive, whereby the ZOI surrounding the cephalosporin +clavulanic acid disk was a minimum of 5 mm diameter larger than the ZOI for the corresponding cephalosporin disk alone for ≥1 antibiotic pair, were continued to the full AST as described. Non-ESBL producing 3GCR isolates which did not demonstrate a typical positive result for ESBL production on the double disk test, but which demonstrated a pattern suggestive of AmpC production whereby there was no, or minimal, ZOI present surrounding the clavulanic acid disk(s), were also continued to full AST.

### Bacterial enumeration

Bacterial enumeration was undertaken for food samples using the Miles and Misra method. An initial suspension was made up to a 1/10 dilution (25 g food in 225 mL BPW) and 1 mL was then added to 9 mL BPW to make a 1/100 dilution. Three 20 μL drops of the 1/100 dilution broth were placed onto a section of a HECA plate, followed by three 20 μL drops of the 1/10 dilution broth onto a separate section. Plates were incubated overnight at 37°C.

Individual blue *E. coli* colonies and rose-pink colonies indicative of other *Enterobacteriaceae* were counted and an average of the counts of the three drops per dilution was calculated, followed by calculating the colony forming units (CFU)/g for each sample.

Bacterial counts were compared to the acceptable levels for laboratory testing of *E. coli* and *Enterobacteriaceae* presence in animal by-products (ABP) as published by Defra (Defra/APHA, 2020) accessed July 2021. For the purpose of the present study, the acceptable reference levels were those presented for one sub-sample tested per sample, where samples would fail if one sub-sample tested had greater than 5,000 CFU/g *E. coli* or *Enterobacteriaceae.*

### Whole genome sequencing with sequence typing and characterization of resistance genes

#### i. *Escherichia coli*

DNA extraction was performed on phenotypically ESBL-producing *E. coli* isolates using the QIAamp® DNA mini kit (Qiagen, Crawley, United Kingdom).

Genomic DNA samples were submitted to the Center for Genomic Research, University of Liverpool for Illumina NEBNext Ultra II FS DNA Library Prep, which was completed following the manufacturer’s protocol. Each library was quantified using Qubit and the size distribution assessed using the Fragment Analyzer. These final libraries were pooled in equimolar amounts using the Qubit and Fragment analyzer data.

The quantity and quality of the pool was assessed by Bioanalyzer and subsequently by qPCR using the Illumina Library Quantification Kit from Kapa (KK4854) on a Roche Light Cycler LC480II according to manufacturer’s instructions.

Following calculation of the molarity using qPCR data, template DNA was diluted to 300pM and denatured for 8 min at room temperature using freshly diluted 0.2 N sodium hydroxide (NaOH) and the reaction was subsequently terminated by the addition of 400 mM TrisCl pH = 8. To improve sequencing quality control 1% PhiX was spiked-in. The libraries were sequenced on the Illumina® NovaSeq 6,000 platform (Illumina®, San Diego, United States) following the standard workflow over 1 lane of an S4 flow cell, generating 2 × 150 bp paired-end reads.

Following sequencing, reads were assembled into contigs using SPAdes and contigs smaller than <200 bps were removed. Quality control (QC) was undertaken on assemblies, and those which passed QC were subject to multi-locus sequence typing (MLST) by submitting locus allele sequences to pubmlst.org. eBURST analysis was performed to group similar isolates based on the sharing of alleles, giving each isolate a e-BURST group assignment.

Gene prediction was carried out using Prokka. Detection of AMR genes was undertaken using Resistance Gene identifier (RGI),^1^ and plasmids were identified using PlasmidFinder and the *Enterobacteriaceae* plasmid marker database.

#### ii. *Salmonella* spp.

DNA extraction and WGS was performed on *Salmonella* spp. isolates by the United Kingdom Health Security Agency, Gastrointestinal Bacteria Reference Unit.

Following DNA extraction, isolates were prepared for sequencing with Nextera XT DNA preparation kits, and sequenced on the Illumina HiSeq 2,500 platform in rapid run mode to produce 100 bp paired-end reads. Trimmomatic v0.40 (Bolger et al., 2014) was used to quality trim fastq reads with bases removed from the trailing end that fell below a PHRED score of 30. The Metric Orientated Sequence Type (most) v1 (Tewolde et al., 2016) was used for sequence type (ST) assignment and serotype was assigned using a combination of the Salmonella MLST database and SeqSero2 (Achtman et al., 2012; Ashton et al., 2016; Zhang et al., 2019). FASTQ sequences were deposited in the National Center for Biotechnology Information (NCBI) Sequence Read Archive under the BioProject accession number PRJNA248792.^2^ AMR determinants were sought using Genefinder v1–5, as previously described (Neuert et al., 2018) and using ResFinder 4.1.^3^ Known acquired resistance genes and resistance-conferring mutations relevant to β-lactams (including carbapenems), fluoroquinolones, aminoglycosides, chloramphenicol, macrolides, sulphonamides, tetracyclines, trimethoprim, rifamycins and fosfomycin, and acquired genes associated with colistin resistance, were included in the analysis (Neuert et al., 2018). β-Lactamase variants were determined with 100% identity using the reference sequences downloaded from the Lahey^4^ or National Center for Biotechnology Information (NCBI)^5^ β-lactamase data resources. Reference sequences for acquired resistance genes were curated from those described in the Comprehensive Antimicrobial Resistance Database^6^ and the ResFinder datasets.^7^ Chromosomal mutations were based on previously published variations in the quinolone-resistance-determining regions (QRDRs) of gyrA, gyrB, parC and parE, which are associated with resistance to quinolones.

## Results

### Online survey

In total, 1831 dog owners completed the online survey (*n* = 915 RMD-feeding, 916 NRMD-feeding), providing dietary information for 3,212 dogs (*n* = 1754 RMD-fed, 1,458 NRMD-fed).

The most popular types of food for dogs fed RMD (*n* = 1754) were pre-prepared raw meat and/or bone diets (78.1%, *n* = 1,369), raw eggs (62.8%, *n* = 1,102) and DIY/home-prepared raw meat and/or bone diets (58.8%, *n* = 1,032), whereas the most popular type of food for dogs fed NRMD (*n* = 1,458) was overwhelmingly cooked commercial complete dry food (91.1%, *n* = 1,326; Table 1).

**Table 1:** Frequency (*n*) and percentage (%) of types of food provided to dogs fed RMD (*n* = 1754) and those fed NRMD (*n* = 1,458).

Type of food	% (*n*)	
	*Raw*	*Non-Raw*
Total	54.6 (1754)	45.4 (1458)
Raw meat and/or bones (pre-prepared diet)	78.1 (1369)	–
Raw eggs	62.8 (1102)	–
Raw meat and/or bones (DIY/home-prepared diet)	58.8 (1032)	–
Dried food items (e.g., pig ears, rawhide chews, dried fish skin)	45.6 (800)	30.5 (444)
Cooked eggs	12.1 (212)	10.4 (152)
Cooked commercial complete dry food	9.6 (168)	91.1 (1326)
Cooked fresh meat and/or bones	8.5 (149)	18.3 (266)
Cooked commercial complete wet food	5.7 (100)	35.3 (513)
Vegetables	3.8 (67)	3.3 (48)
Fruit	2.4 (42)	0.9 (13)
Miscellaneous	2.0 (35)	1.3 (19)
Dairy	1.3 (23)	0.7 (10)
Oily fish	1.3 (23)	1.6 (23)
Vegetarian diet	1.0 (18)	2.8 (41)
Leftovers	0.9 (15)	0.9 (13)
Cold pressed food	0.5 (8)	0.3 (5)
Fresh fish	0.5 (8)	–
Bone broth	0.3 (6)	0.1 (2)
Dehydrated meat	0.3 (6)	–
Frozen fish	0.3 (6)	–
Liver	0.3 (6)	0.1 (1)
Rabbit ears	0.3 (6)	–
Raw fish	0.3 (6)	–
Mussels	0.2 (3)	–
Air dried raw	0.1 (1)	–
Dehydrated offal	0.1 (2)	–
Fish	0.1 (2)	0.2 (3)
Freeze dried raw	0.1 (1)	0.1 (2)
Green tripe	0.1 (1)	–
Home cooked	0.1 (1)	0.3 (5)
Hooves	0.1 (1)	–
Whole prey	0.1 (1)	–
Starchy carbohydrates	–	0.6 (9)

Both food types included in the survey as multiple-selection answers and those detailed additionally as free text answers by dog owners within the ‘other’ category are listed.

The main sources of food provided to dogs fed RMD were shop bought, pre-prepared frozen raw food (55.1%, *n* = 966), raw food from an online supplier (48.2%, *n* = 846) and fresh raw meat from the butcher or supermarket (41.2%, *n* = 723). The main source of food for dogs fed NRMD was shop bought or purchased online cooked dry kibble (84.6%, *n* = 1,233; Table 2). The predominant sources of non-pre-prepared raw meat for those who fed RMD were the supermarket (38.4%, *n* = 673) and butcher (37.8%, *n* = 663) (Appendix Table A1).

**Table 2:** Frequency (*n*) and percentage (%) of sources of the food provided to dogs fed RMD (*n* = 1754) diet and those fed NRMD (*n* = 1,458) diet.

Source	% (*n*)	
	*Raw*	*Non-Raw*
Total	54.6 (1754)	45.4 (1458)
Shop bought, pre-prepared, frozen raw food	55.1 (966)	–
Raw food from an online supplier	48.2 (846)	–
Fresh raw meat from the butcher or supermarket	41.2 (723)	–
Fresh raw meat from another source, e.g., specialist raw meat diet shop	29.1 (511)	–
Shop bought, pre-prepared, fresh raw food	9.9 (173)	–
Shop bought or purchased online cooked dry kibble	9.0 (157)	84.6 (1233)
Shop bought or purchased online, pre-prepared fresh cooked food, e.g., tins, trays, sachets	5.8 (102)	32.6 (475)
Fresh meat from butcher or supermarket, but cook it before feeding	5.3 (93)	12.3 (179)
Shop bought or purchased online, pre-prepared frozen cooked food	4.0 (71)	3.6 (52)
Fresh meat from another source, but cook it before feeding	0.6 (10)	2.7 (40)
Abattoir	0.3 (5)	–
Farmers	0.1 (2)	–
Fishmonger	0.1 (2)	0.3 (4)
Game	0.5 (9)	–
Ourselves	0.1 (2)	0.5 (8)
Roadkill	0.1 (1)	–
Specialist supplier	0.3 (6)	–
Trainer	–	0.2 (3)
Vets	–	2.1 (30)

The table is split into three sections which detail the sources most commonly offered to RMD-fed dogs, the sources most commonly offered to dogs fed NRMD and miscellaneous sources which were provided as additional or alternative ‘other’ sources by dog owners using the associated free text box provided in the survey.

The most commonly fed types of raw meat provided to RMD-fed dogs (*n* = 1754) as either a pre-prepared commercial raw diet or part of a non-pre-prepared DIY/home-prepared meal were offal (83.0%, *n* = 1,456), beef (82.6%, *n* = 1,448), lamb (79.0%, *n* = 1,386), chicken (78.2%, *n* = 1,372), turkey (75.0%, *n* = 1,315) and duck (72.8%, *n* = 1,277; Table 3). Types of raw meat that were represented at less than 2% were excluded.

**Table 3:** Frequency (*n*) and percentage (%) of types of meat provided to dogs fed RMD (*n* = 1754), either as part of pre-prepared commercial raw diet or non-pre-prepared meat (meat types represented at <2% were excluded).

Type of meat	% (*n*)
Total	1754
Offal (e.g., Tripe, heart, liver, kidney)	83.0 (1456)
Beef	82.6 (1448)
Lamb	79.0 (1386)
Chicken	78.2 (1372)
Turkey	75.0 (1315)
Duck	72.8 (1277)
Rabbit	65.2 (1143)
Venison	61.4 (1077)
Game (e.g., Pheasant, grouse, pigeon)	47.5 (834)
Pork	44.7 (784)
Fish	7.7 (135)
Goat	3.4 (59)
Kangaroo	2.9 (51)
Oily fish	2.8 (49)
Horse	2.6 (45)

The preferred types of treats given to dogs fed RMD and to those fed NRMD are detailed in Appendix Table A2. The most popular types of treat for dogs fed RMD were freeze-dried meat/fish treats (56.8%, *n* = 997), raw bones (56.2%, *n* = 986) and dried treats such as chicken feet, pig ears and rawhide (55.5%, *n* = 973). By far, the most popular type of treat for dogs fed NRMD was shop bought cooked treats/biscuits (78.7%, *n* = 1,148).

#### Packaging materials and traceability information available on sample packs

**Figure 1:**
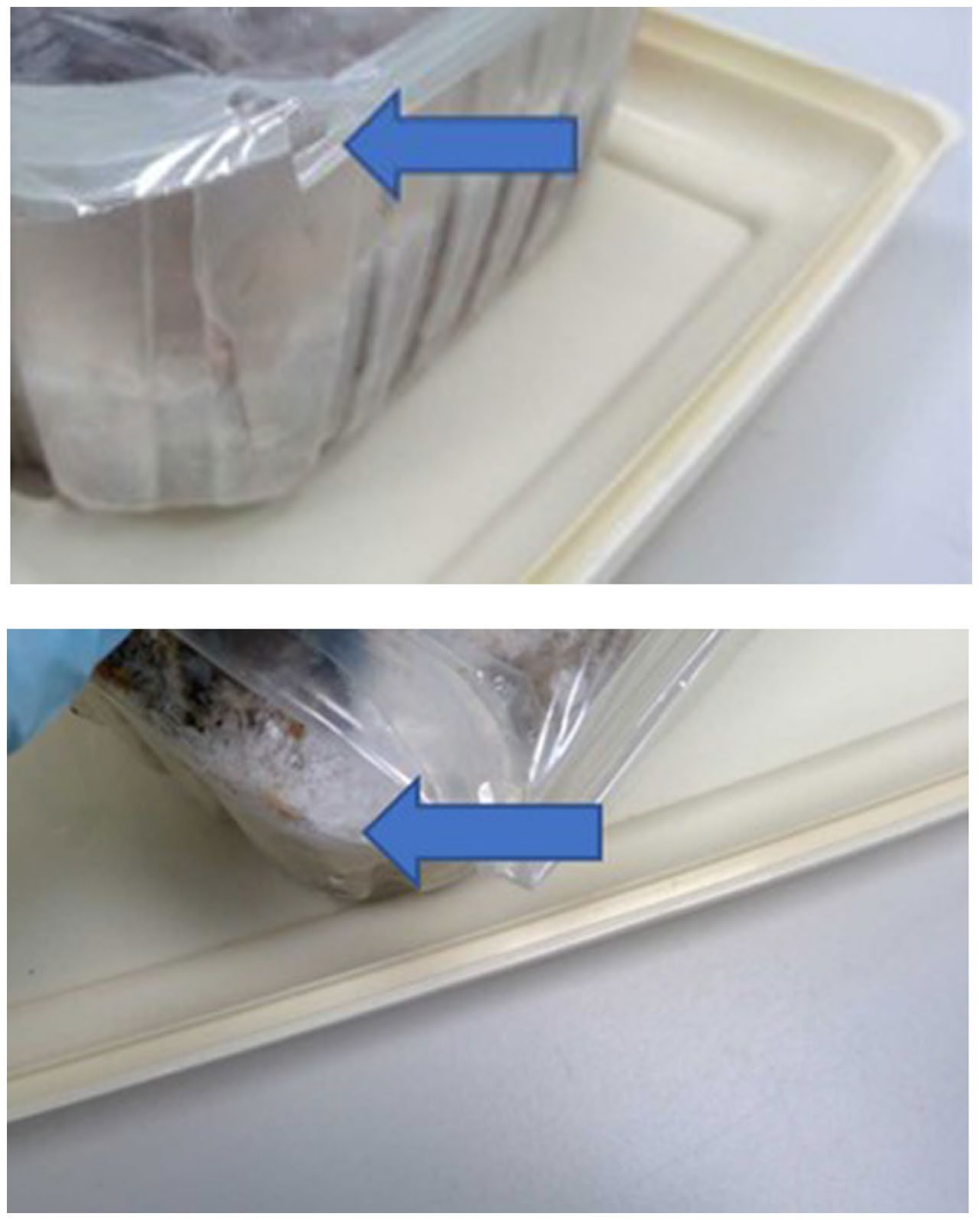
Shattered rigid plastic packaging and open film seal from samples from two different brands of RMD.

**Figure 2:**
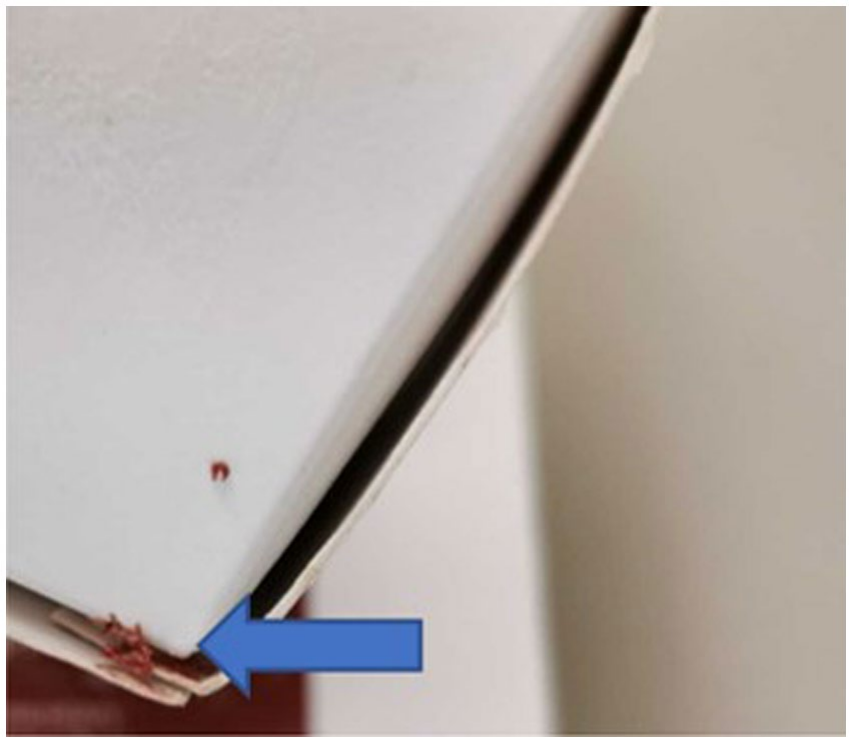
Frozen raw material evident on outside of cardboard packaging of RMD sample prior to defrosting.

**Figure 3:**
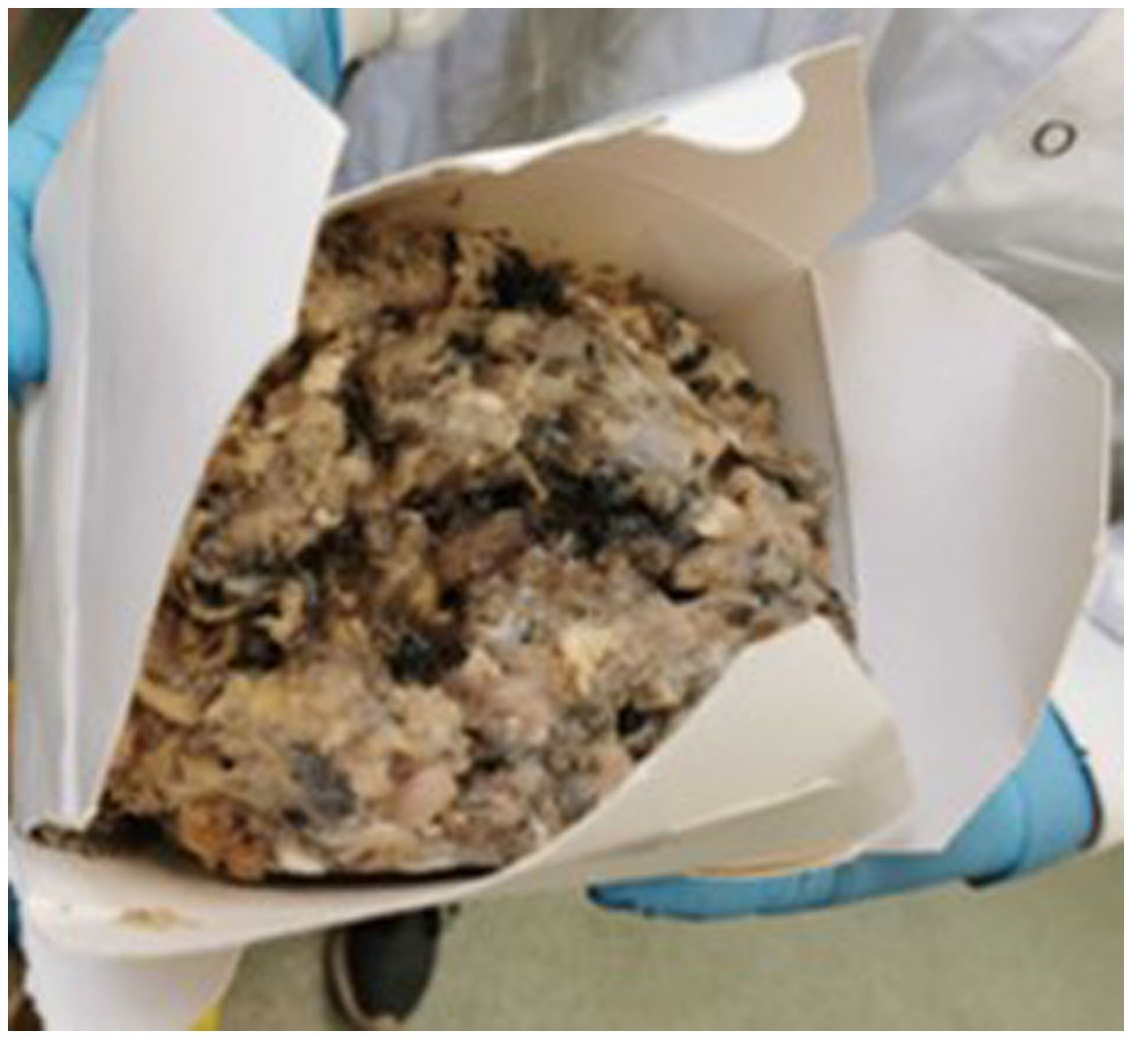
Disintegrated cardboard packaging following defrosting of a RMD sample.

**Figure 4:**
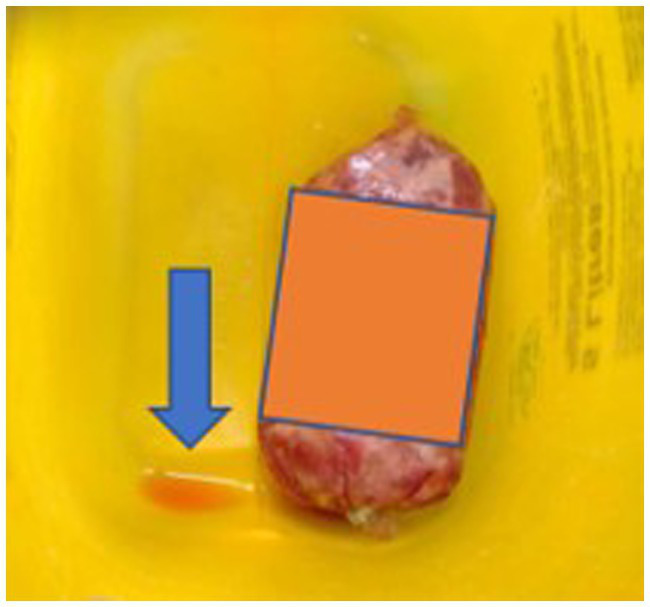
Leaked bloody fluid following defrosting of a RMD sample.

Of the 10 RMD brands studied, 6 had batch numbers present on the sample packs, although it was not always clearly stated as some were present on sticky labels which became loose, or had printed numbers on the packets which were presumed to be batch numbers, although were not explicitly labeled as such (Appendix Table A3). Five brands clearly stated that the meat ingredients were sourced from the United Kingdom, and five had an unknown meat source but terminology such as ‘organic’ and ‘ethically sourced’ were used instead (Appendix Table A3). Whether the products were made in the United Kingdom was not clear for all brands, and only two brands stated specifically that they were made in the United Kingdom, however others stated they used British ingredients or used terminology such as ‘packed in the United Kingdom. The sample packs themselves were not swabbed for evidence of contamination, however samples from four brands were damaged on arrival and as such did not have sealed contents, and samples from eight brands did not have leakproof packaging. Samples from two brands were presented in cardboard packaging which subsequently became compromised on defrosting. Figures 1–3 demonstrate some of the damaged and contaminated packaging observed in this study. Figure 4 demonstrates fluid leakage in the bottom of a defrosting box following defrosting of a sample from one brand tested.

### Laboratory analysis

#### Enumeration of *Escherichia coli* and other *Enterobacteriaceae*

Enumeration of *E. coli* and other *Enterobacteriaceae* was undertaken on 110 RMD samples and 24 NRMD samples from 10 brands each (Table 4, full enumeration results presented in Appendix Table A4). No bacteria were isolated from any of the NRMD samples.

**Table 4:** Bacterial enumeration from RMD samples, illustrating the number of samples per brand tested, the number of samples with >5,000 CFU/g of *E. coli* and other Enterobacteriaceae, the maximum CFU/g of *E. coli* and other *Enterobacteriaceae* isolated within a sample from each brand, and the RMD ingredients within the sample associated with this count.

Anonymised brand	*N* samples	*N* samples with > 5,000 CFU/g *E. coli*	Maximum sample count (CFU/g)	Protein type	*N* samples with > 5,000 CFU/g other *Enterobacteriaceae*	Maximum sample count (CFU/g)	Protein type
B1	13	3	3.0×10^4^	Beef	5	8.7×10^3^	Chicken, tripe
B2	15	3	3.8×10^4^	Offal	4	1.5×10^4^	Beef
B3	14	3	2.57×10^5^	Lamb	3	1.05×10^5^	Lamb
B4	9	0	1.0×10^3^	Beef	2	6.7×10^4^	Goat
B5	10	2	6.2×10^4^	Pork, chicken	4	2.8×10^5^	Pork, chicken
B6	9	4	2.9×10^5^	Beef, offal	3	6.0×10^4^	Tripe, heart
B7	10	6	4.7×10^5^	Pigeon with feather	7	2.0×10^5^	Pork, chicken
B8	10	1	5.5×10^3^	Turkey	0	2.7×10^3^	Turkey
B9	10	1	1.1×10^5^	Chicken	3	1.7×10^5^	Chicken
B10	10	4	3.4×10^5^	Lamb tripe	3	9.8×10^4^	Beef

Among RMD samples, 24.5% (27/110) had counts for *E. coli,* and 30.9% (34/110) had counts for other *Enterobacteriaceae*, greater than 5,000 CFU/g, and therefore would fail Defra testing. Additionally, 20.0% (22/110) of samples had counts of both *E. coli* and other *Enterobacteriaceae* present within the same sample which each exceeded 5,000 CFU/g. Of the brands tested, 80% (8/10) had at least one sample tested which had counts of both *E. coli* and other *Enterobacteriaceae* greater than 5,000 CFU/g, and for one brand, 60% (6/10) of samples tested had *E. coli* counts greater than 5,000 CFU/g, and 70% (7/10) had counts of other *Enterobacteriaceae* greater than 5,000 CFU/g. The highest CFU/g for *E. coli* was associated with minced feathered pigeon, and the highest CFU/g for other *Enterobacteriaceae* was associated with a pork and chicken mix.

#### Antimicrobial susceptibility

The majority of tested RMD samples grew *E. coli* (99.1%, 109/110) and isolates with phenotypic resistance to at least one class of antibiotic were isolated from 39.1% (43/110) of samples (Table 5). Fluoroquinolone (ciprofloxacin)-resistant *E. coli* was present in 8.2% of samples, and multidrug-resistant (MDR) *E. coli* was isolated from 7.3% (8/110) of samples. No resistance to tigecycline or meropenem was observed.

**Table 5:** Percentage (%) and number (*N*) of RMD samples with resistance to antibiotics, an antimicrobial-resistant (AMR) phenotype, and a multidrug-resistant (MDR) phenotype detected, and their associated component protein ingredients.

Antibiotic	% (*N*) RMD samples with at least one resistant *E. coli* detected	Component protein(s)
Total samples	110	
Ampicillin	30.0 (33)	Lamb, chicken, fish, turkey, offal, tripe, goat, duck
Amoxycillin-clavulanic acid	1.8 (2)	Beef
Ciprofloxacin	8.2 (9)	Chicken, fish, goat, turkey, goose, duck
Tigecycline	0.0 (0)	N/A
TMS*	14.5 (16)	Chicken, fish, offal, tripe, turkey, goat, beef
Amikacin	5.5 (6)	Chicken, offal, tripe, fish, game, lamb
Meropenem	0.0 (0)	N/A
Resistant phenotype	39.1 (43)	Chicken, lamb, fish, turkey, offal, tripe, goat, duck, beef, goose
MDR	7.3 (8)	Goat, turkey, chicken

% = percentage; N/A = non-applicable; *TMS, Trimethoprim-sulphamethoxazole; MDR, Resistance to ≥ 3 antibiotic classes.

AMR *E. coli* was isolated from a number of different meat proteins, while MDR *E. coli* was isolated from samples containing goat, turkey and chicken only.

3GCR-*E. coli* (including ESBL-producing and non-ESBL producing *E. coli*) was identified from 16.4% (18/110) of samples of RMD, and phenotypic ESBL-producing *E. coli* (as determined by a positive double-disk test result) was isolated from 13.6% (15/110) of samples (Table 6).

**Table 6:** Antimicrobial resistances observed among 3GCR-*E. coli* iisolates from RMD samples-aggregated sample level data.

Brand	Sample ID	Component protein(s)	ESBL- *E. coli*	3GCR-*E. coli*	MDR- *E. coli*	Antibiotic resistance Profile (S/R)*								
						Amp	Amxc	Cip	Tig	TMS	Ami	Mer	Ctx	Ctz
B1	4	Chicken, tripe	Y	Y	Y	R	S	S	S	R	S	S	R	R
	8	Chicken, tripe	Y	Y	Y	R	S	S	S	S	R	S	S	R
	12	Chicken, tripe	Y	Y	N	R	S	S	S	S	S	S	S	R
	13	Offal, salmon	Y	Y	N	R	S	S	S	S	S	S	R	R
B2	3	Duck	N	Y	Y	R	R	S	S	R	S	S	R	R
	11	Duck	Y	Y	N	R	S	S	S	S	S	S	R	S
	13	Beef	Y	Y	Y	R	S	R	S	S	S	S	R	R
B3	5	Duck	Y	Y	N	R	S	S	S	S	S	S	R	S
	8	Lamb	Y	Y	N	R	S	S	S	S	S	S	R	R
	12	Game, tripe	Y	Y	Y	R	S	R	S	R	S	S	R	R
	13	Beef, tripe	Y	Y	N	R	S	S	S	S	S	S	R	R
B4	1	Goat	Y	Y	Y	R	S	R	S	R	S	S	R	S
	5	Goat	Y	Y	Y	R	S	R	S	R	S	S	R	S
B5	1	Duck	N	Y	Y	R	R	S	S	S	S	S	R	R
	7	Duck	N	Y	Y	R	R	S	S	S	S	S	R	R
B6	1	Lamb	Y	Y	Y	R	S	R	S	R	S	S	R	R
	2	Chicken, beef, lamb, tripe, offal	Y	Y	Y	R	S	R	S	R	S	S	R	S
B7	1	Pork, chicken	Y	Y	N	R	S	S	S	S	S	S	R	S

*Amxc, Amoxycillin-clavulanic acid; Amp, Ampicillin; Tig, Tigecycline; TMS, Trimethoprim sulphamethoxazole; Ami, Amikacin; Cip, Ciprofloxacin; Mer, Meropenem; Ctx, Cefotaxime; Ctz, Ceftazidime. S, susceptible; R (orange boxes), resistant.

From the 18 samples yielding 3GCR-*E. coli*, antimicrobial resistances were identified in the following proportions: Ampicillin 100% (*n* = 18), amoxycillin-clavulanate 17% (*n* = 3), ciprofloxacin 33% (*n* = 6), TMS 39% (*n* = 7), cefotaxime 89% (*n* = 16), ceftazidime 67% (*n* = 12). No resistance to tigecycline or meropenem was observed.

MDR-ESBL-producing *E. coli* was isolated from 10.0% (11/110) of samples, and fluoroquinolone resistant ESBL-producing *E. coli* was isolated from 5.5% (6/110). Resistances to ciprofloxacin and to TMS were observed in 40% (6/15) of samples with ESBL-producing *E. coli* present.

Of the samples where ESBL-producing *E. coli* was present, 46.7% (7/15) included tripe and/or offal as a component ingredient, and 33.3% (5/15) were composed, at least in part, of chicken. 3GCR-*E. coli* was most frequently isolated from samples containing offal/tripe (38.9%, 7/18) and duck (27.8%, 5/18) (Appendix Table A6).

#### Whole genome sequencing results

3GCR-*E. coli* isolates which demonstrated ESBL-production on the double disk test, or were non-ESBL producing and suspected of having AmpC, and which demonstrated a unique resistance phenotype within a sample underwent WGS (*n* = 17) Of these, 13 were phenotypic ESBL-producing *E. coli*, as determined by the double-disk test, and four were suspected to have their ESBL phenotype ‘masked’ due to the presence of pAmpC genes. Representative isolates were sent from all food samples except brand B4 as isolates were not available.

Eleven distinct sequence types (STs) were identified. The most frequently observed ST was ST10 (*n* = 4). Food samples with ST10 contained duck, lamb, beef, tripe, pork and chicken. Other STs represented by more than one isolate were ST58, ST69 and ST1629 (*n* = 2 for each). There was no distinct relationship between the food protein types and the STs observed, other than for ST1629 where both *E. coli* isolates were from a combined chicken and tripe product (Table 7).

**Table 7:** Food protein, sequence type, phenotypic antimicrobial resistance as determined by disk diffusion and resistance genes present for ESBL-producing/3GCR- *E. coli* isolates from raw food samples.

			Antimicrobial resistance genes and their variants*												Phenotypic resistance						
Isolate ID	Meat protein (s)	ST	*ctx-m*	*tem*	*shv*	*cmy*	*qnr*	*gyrA^*	*parC^*	*tet*	*sul*	*dfrA*	Aminoglycoside resistance genes	Chloramphenicol resistance genes	Amp	Amxc	Cip	TMS	Ami	Ctx	Ctz
F92	Duck	10	1		7										R	S	S	S	S	R	S
F104	Lamb	10	15	216											R	S	S	S	S	R	R
F118	Beef, tripe	10	15	216											R	S	S	S	S	R	R
F199	Pork, chicken	10								B, R			*aph(3″)-Ib, aph(6)-Id*		R	S	S	S	S	R	S
F9	Chicken, tripe	48	15	1			S1			A, M	2, 3	12, 14	*aadA2, ant(3″)-Iia, aph(3″)-Ib, aph(6)-Id*	*cmlA6*	R	S	S	R	S	R	R
F68	Duck	58	55				S1						*aac(3)-Iid, aph(3″)-Ib*		R	S	S	S	S	R	S
F184	Lamb	58	15, 107				S1								R	S	R	R	S	R	R
F157	Duck	69		1		2				A					R	R	S	S	S	R	R
F185	Chicken, beef, tripe, lamb, offal	69	27					Yes	Yes	A	2	17	*aadA5,aph(3″)-Ib,aph(6)-Id*		R	S	R	R	S	R	S
F154	Duck	155				2				A					R	R	S	S	S	R	R
F113	Game, tripe	542	15	1			S1			A, B, R	2	14	*aph(3″)-Ib, aph(3′)-Ia, aph(6)-Id*		R	S	R	R	S	R	R
F57	Duck	602				2									R	R	S	S	S	R	R
F11	Chicken, tripe	1,629		52						A	2		*aph(3″)-Ib, aph(6)-Id*		R	S	S	S	R	S	R
F33	Chicken, tripe	1,629		52						A	2		*aph(3″)-Ib, aph(6)-Id*		R	S	S	S	S	S	R
F36	Offal, salmon	4,096	15				S1								R	S	S	S	S	R	R
F80	Beef	4,681	15				S1								R	S	R	S	S	R	R
F56	Duck	6,958		1		2					2	14	*aph(3″)-Ib, aph(6)-Id*	*catB9*	R	R	S	R	S	R	R

ST, Sequence type. Amp, ampicillin; Amxc, amoxycillin-clavulanic acid; Ami, amikacin; Cip, ciprofloxacin; TMS, Trimethoprim-sulphamethoxazole; Ctx, cefotaxime; Ctz, ceftazidime. S, susceptible; R (orange boxes), resistant. No resistance to meropenem or tigecycline was observed, so these have been omitted from this table. *Gene variant columns contain the letter or number for the assigned gene variant identified in an isolate by WGS. ^Yes: indicates a mutation present associated with resistance in that gene.

Multiple AMR genes were identified (Table 7). In terms of ESBL-encoding genes, *bla*_CTX-M_ genes were present in 10 isolates (59%). The most frequently identified was *bla*_CTX-M-15_, present in seven isolates (41%), which were associated with a range of STs. The *bla*_CTX-M-1_ gene was identified in one isolate, which was ST10. The *bla*_CTX-M-27_ and *bla*_CTX-M-55_ genes were present in one isolate each (ST69 and ST58, respectively). One isolate, which was ST58, carried both *bla*_CTX-M-15_ and *bla*_CTX-M-107_ genes. *bla*_TEM_ genes were identified in 47% (8/17) of isolates, however the only ESBL-encoding variant isolated was *bla*_TEM-52_, which was identified in two isolates (both ST 1629). The ESBL-encoding *bla*_SHV-7_ gene was identified in one isolate (ST10) only. The *bla*_OXA_ gene was not observed in any of the isolates. Five isolates did not have *bla*_CTX-M_, *bla*_TEM_ or *bla*_SHV_ ESBL genes present, however four of these did have the AmpC gene *bla*_CMY-2_ present (Table 7). These isolates were 3GCR, and demonstrated phenotypic amoxycillin-clavulanic acid resistance on AST.

Of the 10 MDR isolates, four were associated with the presence of *bla*_CTX-M-15_, and were ST48, ST58, ST542 and ST4681. The isolates were associated with raw food samples containing chicken (*n* = 1), tripe (*n* = 2), lamb (*n* = 1), game (*n* = 1) and beef (*n* = 1). The *qnrS1* gene, associated with quinolone resistance, was present in 35% (6/17) of isolates. Of these, five isolates were associated with concurrent presence of *bla*_CTX-M-15_ and one isolate was associated with concurrent *bla*_CTX-M-1_. However, only three of the isolates which carried the *qnrS1* gene demonstrated phenotypic fluoroquinolone resistance. Additionally, one ST69 isolate which demonstrated phenotypic fluoroquinolone resistance carried both *gyrA* and *parC* gene variants, alongside concurrent *bla*_CTX-M-27_. In terms of trimethoprim-sulphamethoxazole (TMS) resistance, the *dfr* gene (trimethoprim resistance) was found in 24% (4/17) of isolates, and the *sul* gene (sulphomethoxazole resistance) in 35% (6/17) of isolates. All isolates which carried the *dfr* gene also carried the *sul* gene, and coincided with phenotypic TMS resistance. Interestingly, two of the isolates (ST48 and ST542) which carried both *dfr* and *sul* genes, and demonstrated phenotypic TMS resistance, also carried *qnrS1* and *bla*_CTX-M-15_.

Multiple genes encoding aminoglycoside modifying enzymes were present, however only one isolate (ST1629) demonstrated phenotypic resistance to amikacin, the test aminoglycoside, where resistance genes *aph(3″)-Ib* and *aph(6)-Id* were present. Additional genes of interest present which were not specifically tested for phenotypic resistance included those encoding chloramphenicol and fosfomycin resistance.

#### Plasmid analysis

Incompatibility (Inc) group plasmids associated with ESBL genes of interest in the ESBL-producing *E. coli* isolates in this study are presented in Appendix Table A7. IncF was the most frequently identified plasmid group (*n* = 7 IncF types). Within this, plasmid type IncFIB was identified most commonly. Multiple IncF plasmids were associated with *bla*_CTX-M-15_ presence, as well as IncH and IncI group plasmids. One isolate carried *bla*_CTX-M-55_ (ST58), and as well as being associated with IncF group plasmids, it was the only isolate associated with plasmid IncX1. Four isolates carried *bla*_CMY-2_, three of which were associated with IncFIB and IncFIC, with the fourth isolate (ST602) being linked to IncI2(Delta) only. All but two of the MDR food isolates were associated with the presence of IncF plasmids. For the two that were not associated with IncF plasmids, one (ST602) was associated with IncI2(Delta), the other did not have an identified Inc. group plasmid present.

#### *Salmonella* spp.

Of the RMD samples, 17.3% (19/110) had turquoise colonies present on CASE agar, indicating presumptive *Salmonella* spp. No presumptive *Salmonella* spp. colonies were isolated from NRMD samples.

Following this, five (4.5%, 5/110) RMD samples from two different brands (brands 2 and 10) were confirmed to have *Salmonella enterica* present (Table 8). There was a diverse range of *S. enterica* serotypes; *S.* Kottbus, *S. typhimurium* (monophasic), *S. Indiana* and *S.* Enteriditis. In addition, a separate subspecies, S. *diarizonae*, was identified.

**Table 8:** Sample number, raw meat protein type, brand, sequence type and *S. enterica* serotype, alongside antimicrobial resistance (AMR) genes identified and susceptibility results of isolates confirmed as *Salmonella* isolated from raw food samples in this study.

Brand	Sample number	Sample type	Sequence type	*Salmonella* Serotype	*SRA accession*	AMR genes					Phenotypic resistance*						
						*TEM*	*gyrA*	*parC*	*sul*	Aminoglycoside resistance genes	Amp	Amxc	Tig	TMS	Ami	Cip	Mer
2	3A	Duck	582	Kottbus	SRR18545465	*TEM-1D*		*parC*[57:T-S]		*aac(6′)-laa, aac(6′)-ly[v]*	R	S	S	S	S	S	S
2	3B	Duck	582	Kottbus	SRR18545474	*TEM-1D*		*parC*[57:T-S]		*aac(6′)-laa, aac(6′)-ly[v]*	R	S	S	S	S	S	S
2	5A	Tripe	34	Typhimurium (monophasic)	SRR18545464	*TEM-1B*			*sul-2*	*aac(6′)-laa, aph(3″)-lb, aph(6′)-ld*	R	S	S	S	S	S	S
2	11A	Duck	17	Indiana	SRR18544748			*parC*[57:T-S]		*aac(6′)-laa, aac(6′)-ly[v]*	S	S	S	S	S	S	S
2	11B	Duck	17	Indiana	SRR18544750			*parC*[57:T-S]		*aac(6′)-laa, aac(6′)-ly[v]*	S	S	S	S	S	S	S
10	2A	Goose	11	Enteritidis	SRR18545461		*gyrA*[83:S-Y]			*aac(6′)-laa, aac(6′)-ly[v]*	S	S	S	S	S	S	S
10	1A	Lamb tripe	432	*diarizonae* (subsp.)	SRR18545463			*parC*[57:T-S]		*aac(6′)-laa, aac(6′)-ly[v]*	S	S	S	S	S	S	S

*Amxc, Amoxycillin-clavulanic acid; Amp, Ampicillin; Tig, Tigecycline; TMS, Trimethoprim sulphamethoxazole; Ami, Amikacin; Cip, Ciprofloxacin; Mer, Meropenem. S, susceptible; R (orange boxes), resistant.

Within each brand, each specific *S. enterica* serotype was associated with a specific food protein type. Two samples which contained duck from brand B2 were separately contaminated with two different serotypes (*S.* Kottbus and *S. Indiana*). *S.* Kottbus and *S. typhimurium* isolates demonstrated resistance to ampicillin on AST, and on WGS were found to harbor *bla*_TEM-1_ genes. Although all isolates harbored the aminoglycoside resistance gene *aac(6′)-laa*, no phenotypic resistance to amikacin was observed. No further phenotypic antimicrobial resistance was observed in any of the isolates.

## Discussion

The present study has provided important information regarding diet choices for pet dogs in the United Kingdom, and contributes to the growing body of evidence regarding the microbiological concerns surrounding RMD. RMD samples were contaminated with potentially pathogenic bacteria with zoonotic potential, and were associated with the presence of *E. coli* which demonstrated resistance to highest priority critically important antibiotics (HPCIAs), which are important in both human and veterinary medicine.

While conventional commercial cooked diets remain the staple diet for the majority of dogs worldwide other choices are increasing in popularity. A survey of pet owners in the United States and Australia identified that home prepared diets, raw food and table scraps comprised approximately a quarter of the diet for 17.3% of dogs, with provision of bones and raw food at least weekly for nearly a quarter of dogs (Laflamme et al., 2008). A more recent survey of dog owners from the United States, Canada, Australia, New Zealand and the United Kingdom also observed that while conventional commercial feeds were provided to the majority of pet dogs, only 13% of dogs were fed conventional commercial feeds exclusively, with many being provided additions of homemade food and/or RMD (Dodd et al., 2020). Additionally, 40.3% of the respondents of a recent internet-based survey of pet food preferences of dog owners in Brazil indicated that they fed RMD, with the majority adopting the diet within the previous year, further suggesting increasing popularity of this diet choice (Viegas et al., 2020). In the present study, approximately 50% of United Kingdom respondents indicated that they fed RMD items at least once per week, which is a higher proportion than reported previously.

While there was a broader range of food types provided to dogs fed RMD than NRMD, the most common type of RMD provided to dogs in the present study was pre-prepared raw meat and/or bones. The more frequent use of pre-prepared diets may reflect the concerns of owners regarding correct diet formulation and the desire to ensure adequate nutrition, but may also reflect convenience, brand familiarity and the increasing use of internet resources and social media for dietary information with the ready use of targeted advertising via these communication streams. Cooked commercial complete dry food was by far the most commonly provided food type for dogs fed NRMD, with >90% of NRMD-fed dogs being provided this as at least a component of the diet. This is a similar finding to previous research (Laflamme et al., 2008; Dodd et al., 2020).

Although over half of the RMD in this study was reportedly purchased frozen from a shop, nearly 50% was also purchased from an online supplier, indicating the importance of internet-based resources. There is a greater availability of products and choice online, and an added convenience of delivery straight to the consumer, and this result echoes the increasing desire for online shopping among people in general (Brand et al., 2020). However, purchasing food through this method could potentially pose further risks as delivery relies on the cold chain remaining uninterrupted, and if disruption or delay occurs at any point the RMD could be exposed to warmer temperatures, thus allowing proliferation of potentially harmful and AMR bacteria. Additionally, packaging may be damaged in transit, resulting in content leakage and contamination of external packaging. This in itself may pose a risk of transmission of potentially harmful bacteria to facilities where the products are stored on defrosting or prepared, and directly to those handling the products. The external packaging of products was not swabbed in the present study; however, this could be a consideration for future research.

The most frequently fed RMD protein sources were offal such as tripe, liver and kidney (83.0%), as well as beef, lamb, chicken, turkey and duck. These results are broadly similar to the study by Morelli et al. (2019) who observed that beef, chicken and turkey were preferred, with type of offal analyzed separately, and Groat et al. (2022), who observed that most dogs fed RMD were fed a mix of meats, with chicken, red meat and tripe being the most frequently chosen. In the present study, approximately half of dogs fed RMD were fed freeze-dried meat/fish, dried foodstuffs such as pig ears, chicken feet and hide and raw bones as treats, which may again echo the desire indicated previously by owners feeding RMD to provide non-processed products which are perceived as ‘more natural’ in general (Bulochova and Evans, 2021b).

*Escherichia coli* was isolated from all but one of the RMD samples tested, however no *E. coli* was cultured from any of the NRMD kibble samples. This is in agreement with recent research from the USA where similarly, no *E. coli* was isolated from samples tested of commercially available conventional diets with no uncooked components (Gibson et al., 2022). Freezing raw meat is often believed to reduce or negate the risks associated with any microbiological contaminants present. However, as demonstrated in this study, this is not the case for *E. coli* and other *Enterobacteriaceae*, or *Salmonella* spp. Importantly, the freezing process did not kill these bacteria.

In the present study, *E. coli* and other *Enterobacteriaceae* were present at >5,000 CFU/g, therefore exceeding Defra/APHA sub-sample acceptable thresholds, in a quarter and a third of RMD samples tested, respectively. Nine out of 10 brands tested had at least one sample tested which had counts of *E. coli* or other *Enterobacteriaceae* which were greater than those deemed acceptable by Defra/APHA. This highlights that pre-prepared RMD samples tested were frequently contaminated with bacteria which can be pathogenic and cause zoonotic disease, often to a concerningly high degree. This finding is in agreement with those of previous studies (Weese et al., 2005). In one Swedish study, bacteria belonging to the family *Enterobacteriaceae*, including *E. coli*, was present at a level which exceeded EU regulations for raw meat intended for pet food production in 52% of RMD samples (Hellgren et al., 2019), and in a study from Switzerland, 73% of samples tested exceeded these limits (Nüesch-Inderbinen et al., 2019). This was also the case for frozen commercially available RMD in Thailand (Kananub et al., 2020) and in Italy, where samples were found to be contaminated with *Salmonella* spp., *E. coli* O157:H7, *Listeria monocytogenes* and *Campylobacter* spp., despite freezing (Bottari et al., 2020). In another study from Italy, RMD products purchased online and received frozen were found to be highly contaminated with *E. coli* immediately following defrosting, as well as having *Listeria* spp., *Clostridia* spp. and *Yersinia* spp. present, and were suggested to be of poor microbiological quality initially, but demonstrated distinct worsening of quality if products were improperly refrigerated, or not utilized immediately following defrosting (Morelli et al., 2020).

All brands of food tested in the present study were received frozen, stored at -20°C, then defrosted overnight in the fridge prior to testing. While some bacterial multiplication could have occurred during the defrosting process, this is unlikely due to the refrigeration throughout, and rapid processing of samples once defrosted. If there were any breaks in the cold chain during the packing and delivery process, this may have allowed bacterial multiplication. However, this mirrors the process by which owners would receive the foods, therefore it is representative of the microbiological quality of the products received by consumers. All packs were received with insulating packing of different types, and some were more successful at keeping foodstuffs frozen than others, with some leakage of package contents identified in some cases. Nevertheless, it is most likely that a high degree of bacterial contamination was already present in the samples, and this highlights the importance of safe storage (refrigeration at 0-4°C) and defrosting processes for these diets. Additionally, it highlights that RMD products may have poor microbiological quality prior to freezing, thus more needs to be done in manufacturing to minimize contamination, both at source by reviewing the raw materials utilized or during the production process. Previous research has demonstrated that dog owners utilize a number of different methods for defrosting and preparing RMD (Bulochova and Evans, 2021a; Morgan et al., 2022), with poor practices regularly employed, potentially indicating some confusion as to appropriate measures for defrosting RMD. Defrosting processes have been demonstrated previously to be important for food safety, and time–temperature abuse has been shown to be an important factor in the increase in bacteria in contaminated raw meat products, thus increasing the risk of foodborne disease (Roccato et al., 2015). Not all brands tested included detailed instructions for safe defrosting of the product on their product packaging. This highlights an area where improvement is needed regarding safe handling of RMD.

It is concerning that RMD were frequently contaminated with AMR *E. coli*. AMR is a global One Health threat; it has been estimated that in 2015, bacterial AMR infections accounted for 33,000 human deaths in the European Union (EU) and European Economic Area (EEA), with the burden highest in those <1 year and > 65 years old (Cassini et al., 2019). A more recent study estimated that in 2019, 4.95 million deaths globally were associated with bacterial AMR, and 1.27 million deaths were directly attributable (Murray et al., 2022). Furthermore, the United Kingdom 2016 Review on Antimicrobial Resistance estimated that without intervention, as many as 10 million human deaths worldwide could be attributable to AMR by 2050 (O’Neill, 2016).

Approximately 16% of RMD samples tested had 3GCR-*E. coli* present, and 14% had ESBL-producing *E. coli* present. Additionally, 10% of samples tested had MDR ESBL-producing *E. coli* present, with phenotypic resistance to TMS and/or ciprofloxacin observed alongside ESBL-production within many of these isolates. AMR *E. coli* was not isolated from any NRMD samples, a finding similar to that of Baede et al. (2017). It is concerning that *E. coli* which demonstrated concurrent phenotypic resistance to both fluoroquinolones and third generation cephalosporins (cefotaxime, ceftazidime) was isolated from approximately 6% of RMD samples. Both of these antibiotic classes are HPCIAs as determined by the World Health Organization (Veterinary Medicines Directorate, 2015; Collignon et al., 2016). The presence of ESBL-producing and 3GCR-*E. coli* was associated most frequently with samples containing offal/tripe and poultry meat (chicken and duck respectively), however there was no distinct link between meat type and the presence of phenotypic fluoroquinolone resistance, with resistance demonstrated in *E. coli* isolated from RMD samples containing a range of proteins. These meat types were often mixed in combinations in the food samples, however there were single-protein samples of goat, lamb and beef.

The prevalence of 3GCR and ESBL-producing *E. coli* in pre-prepared RMD samples in the present study is lower than that previously reported by smaller studies in Europe. A study of 51 samples of RMD available in Switzerland observed that approximately 61% of samples tested had ESBL-producing *E. coli* present, with the majority of affected samples involving products of cattle or poultry origin (Nüesch-Inderbinen et al., 2019). An additional smaller study of 35 samples from eight brands available in The Netherlands reported that 80% of RMD samples had ESBL-producing *E. coli* isolated (Van Bree et al., 2018). Finally, a study from Sweden identified that 23% of 39 samples tested had 3GCR- *E. coli* present (Nilsson, 2015), and all of the 3GCR *E. coli* harbored the *bla*_CMY-2_ gene, which was also the most frequently observed *bla*_CMY_ gene in the present study.

There remains limited evidence currently regarding the AMR risks specifically from pre-prepared RMD available in the United Kingdom and elsewhere for comparison. However, there are studies of AMR- *E. coli* contamination in meats destined for the human food chain and for pet food. One national study of meat samples purchased from retailers for human consumption in the United Kingdom identified that 65% of chicken samples had ESBL-producing *E. coli* present (Day et al., 2019), another more localized study identified ESBL-producing *E. coli* in 18% of meat products from United Kingdom supermarkets, with the majority of products being chicken (Ludden et al., 2019). While the majority of products were of United Kingdom origin, products were also imported from a range of other countries, highlighting the multinational origin of meat products entering both the human and pet food chains. Finally, a study from Italy identified ESBL-producing and MDR *E. coli* in meat products that were originally packaged at a mass retailer for human consumption but became pet foods once they were deemed no longer suitable for human consumption (Bacci et al., 2019). It is important to note that meat sold for human consumption would be intended to be cooked, which would mitigate the risk of AMR-bacteria (James et al., 2021). A concern regarding DIY/home-prepared raw diets is that the meats used are still likely to harbor zoonotic and AMR bacteria, whereas pre-prepared RMDs should undergo testing to ensure bacteria do not exceed acceptable levels; it is not possible to measure the risk posed by meats from unknown sources prepared within the home. However, it could also be argued that there is potential for more opportunity for cross-contamination within pre-prepared diets in the manufacturing process, particularly in minced products where more than one protein type is included.

In the present study, while a United Kingdom origin for meat ingredients was stated on the sample packets for 50% of the RMD brands tested, the remainder did not specifically state the country of origin of the meats used. Additionally, 60% of the brands tested had a batch number clearly present on the sample packets, but whether the food was produced in the United Kingdom was not clear for a number of brands. This is a concern because it would seem that there is a lack of traceability and provenance of product present, which would prove an issue if there was an outbreak of disease potentially associated with the product. The importance of traceability of RMD ingredients was highlighted when raw hare meat intended for use in RMD was found to be contaminated with *Brucella suis*, and had been imported into the United Kingdom from The Netherlands after originating in Argentina (Frost, 2017). Therefore, this is potentially an area of RMD production which requires attention.

On whole genome sequencing, a number of sequence types (STs) were identified in ESBL-producing *E. coli* isolates, with the most frequently encountered being ST10. ST10 *E. coli* is frequently associated with ESBL-genes, in particular, *bla*_CTX-M_ genes (Oteo et al., 2009; Cormier et al., 2019). Other STs of interest which were identified in ESBL-producing *E. coli* isolated from RMD samples in this study were ST58, ST69 and ST155. *E. coli* ST58 and ST69 are globally disseminated uropathogens and have previously been associated with *bla*_ESBL_ and AMR gene carriage (Novais et al., 2013; De Souza da-Silva et al., 2020; Reid et al., 2022). *E. coli* ST58 has been isolated from livestock and food sources previously (Reid et al., 2022), including raw pet food (Nüesch-Inderbinen et al., 2019). *E. coli* ST155 is an important ExPEC (extraintestinal pathogenic *E. coli*) strain with zoonotic potential, previously identified in beef cattle faces, chicken meat and human blood in the United Kingdom, as well as in RMD samples (Ludden et al., 2019; Nüesch-Inderbinen et al., 2019).

A variety of AMR genes were identified in the ESBL-producing *E. coli* isolates from RMD samples. The predominant *bla*_ESBL_ gene identified was *bla*_CTX-M-15_, present in 41% of isolates. Presence of the *bla*_CTX-M-15_ gene was frequently observed alongside co-carriage of additional plasmid-mediated resistance genes such as *qnrS1*, which mediates fluoroquinolone resistance, and genes encoding resistance to other antibiotic classes such as tetracyclines, trimethoprim-sulphamethoxazole and aminoglycosides. The predominance of *bla*_CTX-M-15_ is of concern as it is carried on mobile transferrable genetic elements which frequently harbor resistance genes to other antimicrobials, including fluoroquinolones, thus increasing the risk of conferring MDR (Baba Ahmed-Kazi Tani et al., 2013). Only one isolate demonstrated the presence of *bla*_CTX-M-1_. Again, there is little data available from pre-prepared RMDs for comparison, however this finding contrasts with the findings of Nüesch-Inderbinen et al. (2019), who observed that *bla*_CTX-M-1_ was the most frequently detected *bla*_ESBL_ gene in ESBL-producing *E. coli* isolated from RMD samples commercially available in Switzerland, although *bla*_CTX-M-15_ was the second-most frequently detected *bla*_ESBL_ gene.

Isolates harboring the *bla*_CTX-M-15_ gene in the present study were not associated with any specific meat protein type. However, the *bla*_CTX-M-15_ gene has also been isolated from ESBL-producing *E. coli* from livestock in the United Kingdom, including from pig meat (Veterinary Medicines Directorate, 2022), and from faces of chicken, beef and pigs (Ludden et al., 2019).

Although *bla*_CTX-M_ genes were the predominant *bla*_ESBL_ genes in this study, *bla*_TEM-52_ was also isolated from two RMD samples containing a combination of chicken and tripe. This ESBL-gene has previously been observed in *E. coli* isolated from United Kingdom produced broiler chickens and turkeys (Randall et al., 2011). Finally, the plasmid-mediated AmpC (pAmpC) resistance gene *bla*_CMY-2_ was identified in four samples, all of which were raw duck, were single-protein, and obtained from two different suppliers. In all cases, isolates were resistant to ampicillin, amoxycillin-clavulanic acid and a third-generation cephalosporin. This gene has been identified in broilers and chicken meat within Europe previously (Voets et al., 2013; Solà-Ginés et al., 2015), and from a ducks in China (Ma et al., 2012; Zheng et al., 2022), however to the authors’ knowledge this is the first report of *bla*_CMY-2_ being present in products containing duck meat in the United Kingdom, although the country of origin of the meat was unknown, again highlighting the importance and need for improved traceability of RMD ingredients.

It is concerning that such a range of AMR genes was present, frequently in combination, within RMDs in the present study. These genes are potentially transmissible through mechanisms such as mobile plasmids and as such these isolates could act as a reservoir for MDR. There are a multitude of RMD brands in the United Kingdom, which utilize meat products sourced from both within the United Kingdom and abroad, therefore larger scale studies are required to investigate the problem with regards to AMR *E. coli* presence in United Kingdom-fed RMDs further, nevertheless the findings of the current study indicate that contamination with AMR *E. coli* is also a problem with RMD fed to dogs in the United Kingdom. This is concerning from an animal health and welfare point of view, but also presents a potential public health risk. Dogs fed RMD have been shown to shed AMR *E. coli* in their faces, and the provision of a raw diet has been demonstrated to be a risk factor for the carriage of ESBL-producing *E. coli* by healthy dogs (Schmidt et al., 2015; Wedley et al., 2017; Runesvärd et al., 2020; Van den Bunt et al., 2020; Groat et al., 2022).

Five (4.5%) RMD samples from two brands were contaminated with *S. enterica*, with five different serotypes/subspecies identified, each associated with a unique meat protein type, however most isolates were susceptible to antimicrobials. The reported prevalence of *Salmonella* spp. contamination in RMD in studies in Europe, Canada and the USA is wide ranging, from 4 to 25% (Weese et al., 2005; Strohmeyer et al., 2006; Mehlenbacher et al., 2012; Nemser et al., 2014; Hellgren et al., 2019; Nüesch-Inderbinen et al., 2019), therefore the prevalence identified in the present study is at the lower end of that range. However, no meat containing *Salmonella* spp. should be present within pet food at retail, and samples which test positive for *Salmonella* spp. at production should be removed from entering sale, therefore the presence of any *Salmonella* spp. contamination is concerning. *S*. Enteriditis and *S. typhimurium* (monophasic) are among the top five serotypes resulting in human infection reported to the United Kingdom Health Security Agency (UKHSA) (Chattaway et al., 2019), however all serotypes present in the RMD samples in the present study have the potential to cause disease in humans, again highlighting the public health concerns associated with RMD. Concerningly, a study of experimentally-inoculated raw meat identified that *Salmonella* spp. persisted in pet food bowls despite standard cleaning methods, including bleach, scrubbing with soap and washing in the dishwasher (Weese and Rousseau, 2006). Furthermore, while much of *Salmonella* spp. contamination occurred in samples containing poultry, *S. enterica* subspecies *diarizonae* was isolated from a sample containing lamb tripe. This is unsurprising as *S. enterica* subsp. *diarizonae* is commonly associated with reptiles and sheep, and although uncommon, invasive human infections with *S. enterica* subsp. *diarizonae* have been reported (Giner-Lamia et al., 2019), demonstrating zoonotic potential. It is noteworthy that dried raw treats were frequently chosen by respondents in present study, however *Salmonella* spp. has also been isolated from dried raw pet treats in the United Kingdom (Morgan et al., 2023).

The presence of *Salmonella* spp. in RMD samples is not only a risk to public health, but it also poses a risk to animal health and welfare. The provision of *Salmonella*-contaminated RMD to pets has been implicated as a cause of mesenteric lymphadenitis in two dogs (Binagia and Levy, 2020), diarrhea and death in Greyhound puppies (Morley et al., 2006), enterocolitis and death in a series of puppies and kittens (Jones et al., 2019) as well as being highly suspected as the cause of two cases of salmonellosis in cats (Giacometti et al., 2017).

## Limitations

There were some limitations to the present study. Approximately 50% of respondents indicated that they fed RMD at least once per week. However, this is unlikely to be representative of the diet choices of the dog owning population in the United Kingdom, with RMD likely being over-represented due in part to the participant recruitment methods and self-selection bias due to a high uptake of the survey within the RMD feeding community, therefore this finding must be interpreted with caution. Nevertheless, this result may be indicative of the interest surrounding RMD and, as in other countries, the popularity of RMD within the United Kingdom is likely to be increasing. Additional research is required to validate this further.

The food brands chosen to test were selected based on the preferences of the dog owners who responded to the survey, not on market share, therefore may not be fully representative of the possible levels of contamination present in dog foods (RMD and NRMD) available in the United Kingdom. There are a multitude of brands available, and different brands which were not sampled may have different levels of contamination. Due to time and financial constraints and product availability at the time of purchase, only a limited number of samples were tested per brand, particularly of NRMD. This may have underestimated the contamination present within a brand, or indeed may have overestimated if a particularly contaminated batch was tested. Additionally, for RMD, bacterial proliferation could have occurred due to a break in the cold chain in transit during the order packing and delivery process, or during defrosting, although this was deemed unlikely as discussed earlier. Furthermore, the present study only tested pre-packaged samples of RMD, and did not include home-prepared/DIY diets, which may have differing levels of contamination as discussed earlier. It also focused on the presence of *Enterobacteriaceae*, and did not screen for other bacteria with zoonotic potential such as *Campylobacter* spp. or *Listeria* spp. There may be an underestimation of the presence of *Salmonella* spp. in the food samples as the method of isolating *Salmonella* spp. using the CASE agar is likely to have selected only for *S. enterica*, which may mean that a small number of other *Salmonella* subspecies could have been missed.

There was a slight deviation from EUCAST AST methods in that the incubator temperature utilized in the present study was 37°C, instead of 35 ± 1°C, however all isolates which were phenotypically ESBL-producing on the double disk test were subjected to WGS for confirmation of the presence of ESBL resistance genes. WGS was only undertaken on 3GCR and ESBL-producing *E. coli* isolates, and further WGS on non-ESBL *E. coli* was beyond the scope of the present study. There may be further resistance genes of interest in the AMR- *E. coli* isolates which were not 3GCR/ESBL-producing, and this warrants further investigation. Furthermore, analysis of *E. coli* and *Salmonella* spp. virulence factors present would provide further depth surrounding the potential health risks associated with RMD. Finally, we were only able to identify the plasmids present which were associated with ESBL gene presence, however further in-depth investigation is required to determine which plasmids genes were carried on specifically, and how transmissible these may be.

## Conclusion

In conclusion, this study has demonstrated a number of concerns surrounding RMDs for dogs available in the United Kingdom. RMD samples were frequently contaminated with fecal bacteria with potential to cause disease in both humans and animals. In addition, there were concerning levels of AMR-*E. coli* present, with resistance to more than one highest priority critically important antibiotic class demonstrated. Furthermore, concerns surrounding packaging damage, leaks and limited traceability in some cases were evident. Therefore, RMD for pets could pose an important human and animal health risk. Pre-prepared RMDs are often sold as ‘human-grade. which may suggest a perceived greater level of quality and safety, however all meats which are utilized within RMDs are graded as at least Defra category 3 ABPs, and as demonstrated, this does not negate the microbiological risks. It is crucial that veterinary professionals, medical staff, pet food retailers and dog owners are aware of these risks, and if dog owners do choose to feed a RMD, it is vital that strict hygiene measures are practiced throughout the food storage, defrosting and preparation processes, including using separate food storage and preparation facilities, practicing thorough hand washing, and disinfection of food bowls and food preparation areas after feeding.

## Data availability statement

The datasets presented in this study can be found in online repositories. The names of the repository/repositories and accession number(s) can be found in the article/Supplementary material.

## Author contributions

GM: Data curation, Formal analysis, Investigation, Supervision, Writing – original draft. GP: Conceptualization, Formal analysis, Funding acquisition, Supervision, Visualization, Writing – review & editing. ET: Data curation, Writing – original draft. MC: Data curation, Formal analysis, Investigation, Methodology, Software, Visualization, Writing – review & editing. VS: Funding acquisition, Supervision, Writing – review & editing. NW: Conceptualization, Funding acquisition, Methodology, Project administration, Supervision, Visualization, Writing – review & editing.

## Footnotes

^1^ https://card.mcmaster.ca/analyze/rgi

^2^ www.ncbi.nlm.nih.gov/bioproject/?term=248792

^3^ https://cge.food.dtu.dk/services/ResFinder/

^4^ www.lahey.org

^5^ www.ncbi.nlm.nih.gov/pathogens/betalactamase-data-resources

^6^ http://arpcard.mcmaster.ca

^7^ https://cge.cbs.dtu.dk/services/data.php
