# Supplementary material for: An investigation of the presence and antimicrobial susceptibility of Enterobacteriaceae in raw and cooked kibble diets for dogs in the United Kingdom
Source: Front Microbiol. 2024 Jan 8;14:1301841. doi: 10.3389/fmicb.2023.1301841 (PMC10800874; doi:10.3389/fmicb.2023.1301841)
Supplement: Supplementary file 1 [file Data_Sheet_1.pdf]

An investigation of the presence and antimicrobial susceptibility of *Enterobacteriaceae* in raw and cooked kibble diets for dogs in the UK: Appendix

Table A1: Frequency (n) and percentage (%) of sources of non-pre-prepared raw food provided to dogs fed RMD (n=1754) diet. Both food sources included in the survey as multiple-selection answers and those detailed additionally as free text answers by dog owners within the 'other' category are listed. Sources represented at <1% were excluded.

Source	% (n)
**Total**	**1754**
Supermarket	38.4 (673)
Butcher	37.8 (663)
Farm Shop	13.3 (234)
Dedicated raw supplier	7.5 (131)
Abattoir	4.9 (86)
Market Stall	3.8 (67)
Online	1.7 (29)
Pet shop	1.7 (29)
Game dealer	1.5 (27)
Hunter	1.3 (22)

Table A2: Frequency (n) and percentage (%) of types of treats provided to dogs fed RMD (n=1754) diet and those fed NRMD (n=1458) diet. Both treat types included in the survey as multiple-selection answers and those detailed additionally as free text answers by dog owners within the 'other' category are listed (indicated by *).

Treats	% (n)	
	Raw	*Non-Raw*
**Total**	**55.0 (1754)**	**45.0 (1458)**
Freeze dried meat/fish treats	56.8 (997)	27.5 (401)
Raw bones	56.2 (986)	-
Dried treats (e.g. pig ears, rawhide, chicken feet)	55.5 (973)	35.0 (510)
Raw meat (including body parts such as feet, hooves)	43.0 (754)	-
Shop bought cooked treats/biscuits	36.5 (640)	78.7 (1148)
Cooked meat	18.1 (318)	27.3 (398)
Homemade treats*	3.9 (68)	2.0 (29)
Dehydrated meat *	3.4 (59)	0.7 (10)
Vegetables *	3.1 (55)	6.5 (95)
Fruit *	2.1 (36)	2.0 (29)
Dairy *	1.9 (33)	2.8 (41)
Dehydrated offal *	1.9 (34)	0.1 (2)
Miscellaneous *	1.9 (33)	1.9 (27)
Liver *	1.5 (27)	1.0 (14)
Oily fish*	1.4 (24)	0.5 (7)

Fish *	1.1 (19)	0.7 (10)
Cooked bones	1.0 (17)	5.6 (81)
Dried/frozen rabbit ears *	0.7 (12)	-
Leftovers *	0.7 (12)	0.8 (11)
Filled bones*	0.3 (5)	-
I don't feed any treats	2.4 (42)	1.7 (83)

Table A3: Presence of batch numbers on packets and source of meats used as ingredients in RMD brands tested in this study, alongside whether the products were made in the UK.

Anonymised Brand	Purchased from	Date Purchased	Batch Number Present	Meat Source	Product Made in UK	Packet material	Pack damaged on arrival	Leakproof packet	Other Information
B1	Direct from brand website	August 2020	No	Unknown	Unknown	Film/thin plastic	No	No	
B2	Direct from brand website	August 2020	Yes	UK	Yes	Plastic tub	Yes	No	
B3	Direct from brand website	September 2020	No	Unknown	States manufactured in Britain	Plastic with film lid	Yes	No	
B4	Direct from brand website	October 2020	Yes	UK	States British ingredients	Plastic tub	Yes	No	Batch number on sticky label for some products, not present on all. Numbers printed on back of some packs but unclear if batch number
B5	Direct from brand website	June 2021	Yes	Unknown	Yes	Cardboard	No	No	Batch number on sticky label. Meat source unknown, states organic and ethically sourced
B6	Direct from brand website	July 2021	No	UK	States packed in UK	Cardboard	Yes	No	
B7	Direct from brand website	September 2021	No	Unknown	Unknown	Plastic with film lid	No	Yes	
B8	Direct from brand website	September 2021	Yes	UK	States British meat	Plastic with film lid	No	Yes	
B9	Direct from brand website	September 2021	Yes	UK	States British meat	Plastic film wrapped with metal clamps on ends	No	No	
B10	Direct from brand website	September 2021	Yes	Unknown	Unknown	Thin flexible plastic with film lid	No	No	

Table A4. Bacterial enumeration results for RMD (n=110 samples). Samples would fail DEFRA testing if they are found to contain bacterial counts of *E. coli* or other *Enterobacteriaceae* greater than 5000 CFU/g.

Brand	Sample number	Flavour	Average *E. coli* CFU/g	Average other *Enterobacteriaceae* CFU/g	Pass *E. coli*? (1 sample tested, fail if >5000 CFU/g)	Pass other *Enterobacteriaceae*? (1 sample tested, fail if <5000 CFU/g)
	1	Lamb with chicken	0	500	Pass	Pass
B1	2	Chicken and salmon	0	1000	Pass	Pass
	3	Beef	26000	7167	**Fail**	**Fail**
	4	Chicken with tripe	1333	8667	Pass	**Fail**
	5	Lamb with chicken	0	333	Pass	Pass
	6	Chicken and salmon	0	333	Pass	Pass
	7	Beef	26667	6333	**Fail**	**Fail**
	8	Chicken with tripe	833	3500	Pass	Pass
	9	Lamb with chicken	0	167	Pass	Pass
	10	Chicken and salmon	0	833	Pass	Pass
	11	Beef	29833	7333	**Fail**	**Fail**
	12	Chicken with tripe	2833	7167	Pass	**Fail**
	13	Mixed offal and salmon	0	1167	Pass	Pass
	1	Tripe	333	167	Pass	Pass
B2	2	Lamb	0	0	Pass	Pass
	3	Duck	0	0	Pass	Pass
	4	Offal	37667	13167	**Fail**	**Fail**
	5	Tripe	4500	3500	Pass	Pass
	6	Turkey	500	667	Pass	Pass
	7	Turkey	0	0	Pass	Pass
	8	Chicken	0	0	Pass	Pass
	9	Boneless beef	3667	8500	Pass	**Fail**
	10	Rabbit	0	0	Pass	Pass
	11	Duck	0	167	Pass	Pass
	12	Offal	21500	12000	**Fail**	**Fail**

	13	Boneless beef	10833	14667	**Fail**	**Fail**
	14	Lamb	0	0	Pass	Pass
	15	Chicken	0	167	Pass	Pass
B3	1	Beef	5667	10000	**Fail**	**Fail**
	2	Chicken	0	0	Pass	Pass
	3	Offal mix	0	0	Pass	Pass
	4	Rabbit	0	0	Pass	Pass
	5	Duck	167	500	Pass	Pass
	6	Game and tripe	167	1000	Pass	Pass
	7	Duck	167	167	Pass	Pass
	8	All lamb	256667	105000	**Fail**	**Fail**
	9	Chicken	0	0	Pass	Pass
	10	Turkey	0	0	Pass	Pass
	11	Chicken and salmon	0	167	Pass	Pass
	12	Game and tripe	167	0	Pass	Pass
	13	Beef and tripe	0	0	Pass	Pass
	14	Lamb	126667	68333	**Fail**	**Fail**
B4	1	Goat	833	40833	Pass	**Fail**
	2	Beef	1000	12167	Pass	**Fail**
	3	Lamb	0	2333	Pass	Pass
	4	Beef	0	667	Pass	Pass
	5	Goat	833	67333	Pass	**Fail**
	6	Lamb	0	1333	Pass	Pass
	7	Chicken	0	833	Pass	Pass
	8	Turkey	0	167	Pass	Pass
	9	Turkey	0	0	Pass	Pass
B5	1	Duck	833	102667	Pass	**Fail**
	2	Beef tripe mince	0	0	Pass	Pass
	3	Chicken (carcass mince)	0	0	Pass	Pass
	4	Pork, Chicken	62167	280000	**Fail**	**Fail**
	5	Turkey	18000	10667	**Fail**	**Fail**

	6	Chicken	1000	2167	Pass	Pass
	7	Duck	1333	17333	Pass	**Fail**
	8	Beef mince	0	667	Pass	Pass
	9	Chicken	833	1167	Pass	Pass
	10	Beef tripe mince	167	0	Pass	Pass
B6	1	Lamb	20000	50000	**Fail**	**Fail**
	2	Chicken, beef, beef tripe, lamb and beef offal	3667	2667	Pass	Pass
	3	Duck	0	0	Pass	Pass
	4	Chicken	0	0	Pass	Pass
	5	Venison	11500	1667	**Fail**	Pass
	6	Chicken	167	0	Pass	Pass
	7	Duck	0	0	Pass	Pass
	8	Tripe and heart	141667	60000	**Fail**	**Fail**
	9	Beef and offal	288333	58333	**Fail**	**Fail**
B7	1	Pork mince with chicken	11000	200000	**Fail**	**Fail**
	2	Duck mince	0	1000	Pass	Pass
	3	Minced pigeon with feather	473333	0	**Fail**	Pass
	4	Lamb, fish with turkey	131667	130000	**Fail**	**Fail**
	5	Ox tripe mince	500	38333	Pass	**Fail**
	6	Tripe and oily fish	1000	13833	Pass	**Fail**
	7	Pig pluck mince boneless	18000	5667	**Fail**	**Fail**
	8	Ox mince boneless	56667	73333	**Fail**	**Fail**
	9	Venison	28333	56667	**Fail**	**Fail**
	10	Chicken mince	333	1667	Pass	Pass
B8	1	80/20 chicken mince	0	1500	Pass	Pass
	2	80/20 turkey mince	5500	1833	**Fail**	Pass
	3	Wild boar and duck	333	333	Pass	Pass
	4	80/20 chicken mince	0	1500	Pass	Pass
	5	80/20 turkey mince	4333	2667	Pass	Pass
	6	80/20 beef and tripe mince	0	167	Pass	Pass
	7	Rabbit and venison	0	0	Pass	Pass

	8	70/30 lamb	833	167	Pass	Pass
	9	Duck and venison	0	0	Pass	Pass
	10	70/30 beef	0	1667	Pass	Pass
B9	1	Free range chicken	110000	170000	**Fail**	**Fail**
	2	Beef	167	0	Pass	Pass
	3	Lamb	500	4833	Pass	Pass
	4	Turkey	0	1500	Pass	Pass
	5	Wild venison	2500	10333	Pass	**Fail**
	6	Free range turkey	0	0	Pass	Pass
	7	Lamb	333	1000	Pass	Pass
	8	Free range duck	0	833	Pass	Pass
	9	Duck	833	17667	Pass	**Fail**
	10	Free range pork	0	0	Pass	Pass
B10	1	Lamb tripe	341667	50000	**Fail**	**Fail**
	2	Minced goose	0	667	Pass	Pass
	3	Lamb and lamb tripe	18167	6500	**Fail**	**Fail**
	4	Beef	500	98333	Pass	**Fail**
	5	Pork	167	1667	Pass	Pass
	6	Lamb 80/10/10	7000	3667	**Fail**	Pass
	7	Free range duck	0	3000	Pass	Pass
	8	Duck and lamb tripe 80/10/10	33167	1500	Fail	Pass
	9	Turkey and lamb	1167	500	Pass	Pass
	10	Chicken and salmon	2500	3667	Pass	Pass

Table A5: Full antimicrobial susceptibility testing results for *E. coli* isolates which underwent whole genome sequencing, including zone of inhibition diameter (mm) and breakpoint (susceptible/resistant, EUCAST 2022)

Sample ID	Antibiotic type																	
	Amp	Amp	Amx	Amx	Cip	Cip	Tig	Tig	TMS	TMS	Ami	Ami	Mer	Mer	Ctx	Ctx	Ctz	Ctz
F92	0	R	23	S	24	S	20	S	25	S	18	S	25	S	9	R	23	S
F104	0	R	21	S	32	S	24	S	32	S	23	S	34	S	0	R	14	R
F118	0	R	22	S	28	S	21	S	26	S	19	S	30	S	0	R	15	R
F199	0	R	24	S	29	S	24	S	31	S	19	S	32	S	8	R	19	S
F9	0	R	21	S	26	S	19	S	0	R	21	S	36	S	0	R	17	R
F68	0	R	25	S	23	S	23	S	32	S	22	S	34	S	9	R	19	S
F184	0	R	23	S	18	R	21	S	0	R	19	S	31	S	9	R	18	R
F157	6	R	9	R	35	S	28	S	32	S	23	S	31	S	11	R	9	R
F185	0	R	25	S	19	R	25	S	0	R	21	S	33	S	11	R	19	S
F154	6	R	8	R	33	S	25	S	30	S	22	S	31	S	12	R	9	R
F113	0	R	19	S	21	R	20	S	0	R	23	S	34	S	0	R	11	R
F57	0	R	10	R	34	S	20	S	28	S	18	S	32	S	13	R	11	R
F11	0	R	21	S	33	S	19	S	19	S	17	R	33	S	18	S	18	R
F33	0	R	20	S	33	S	19	S	19	S	18	S	33	S	19	S	18	R
F36	0	R	22	S	26	S	21	S	27	S	18	S	30	S	1	R	16	R
F80	0	R	19	R	20	R	21	S	29	S	19	S	31	S	0	R	9	R
F56	0	R	9	R	34	S	22	S	0	R	19	S	32	S	10	R	8	R

*Amx: Amoxycillin-clavulanate; Amp: Ampicillin; Tig: Tigecycline; TMS: Trimethoprim sulphamethoxazole; Ami: Amikacin; Cip: Ciprofloxacin; Mer: Meropenem. S: susceptible; R: resistant

Table A6: Percentage (%) and number (n) of RMD samples with ESBL-producing and 3GCR *E. coli* present, and the associated food protein types

Protein type	% (n) samples ESBL- *E. coli*	% (n) samples 3GCR- *E. coli*
**Total**	**15**	**18**
Offal/Tripe	46.7 (7)	33.3 (6)
Chicken	31.3 (5)	22.2 (4)
Beef	18.8 (3)	16.7 (3)
Lamb	18.8 (3)	16.7 (3)
Duck	12.5 (2)	27.8 (5)
Goat	12.5 (2)	11.1 (2)
Fish	6.3 (1)	5.6 (1)
Game	6.3 (1)	5.6 (1)
Pork	6.3 (1)	5.6 (1)
Pigeon	0.0 (0)	0.0 (0)
Rabbit	0.0 (0)	0.0 (0)
Turkey	0.0 (0)	0.0 (0)
Venison	0.0 (0)	0.0 (0)

Other	0.0 (0)	0.0 (0)

Table A7: Inc group plasmids associated with STs and ESBL genes of interest from ESBL-producing *E. coli* isolated from raw dog food

ESBL gene	Gene number	STs associated	Plasmids associated
bla$_{CTX-M}$	1	10	IncI1-I(gamma)
	15	10, 48, 542, 4096, 4681	IncFIA(HI1), IncFIA, IncFIB, IncFIB(K), IncFIC(FII), IncFII(pCoo), IncFII|AY458016, IncHI1A, IncHI1B(R27), IncI1-I(gamma)
	27	69	IncFIA, IncFIB, IncFIC(FII)
	55	58	IncFIA, IncFIB, IncFII, IncX1
bla$_{TEM}$	52	1629	IncFII, IncI1-I(gamma), IncX1, IncY
bla$_{SHV}$	7	10	IncI1-I(gamma)
bla$_{CMY}$	2	69, 155, 602, 6958	IncB/O/K/Z, IncFIA, IncFIB, IncFIC(FII), IncI1-I(gamma), IncI2(Delta)
